# Transgenerational Herbivory Effects on Performance of Clonal Offspring of the Invasive Plant *Alternanthera philoxeroides*

**DOI:** 10.3390/plants12051180

**Published:** 2023-03-04

**Authors:** Qiu-Yue Fu, Cheng-Ling Yu, Ran Dong, Juan Shi, Fang-Li Luo, Jun-Qin Gao, Hong-Li Li, Bi-Cheng Dong, Fei-Hai Yu

**Affiliations:** 1 School of Ecology and Nature Conservation, Beijing Forestry University, Beijing 100083, China; 2 Institute of Wetland Ecology & Clone Ecology/Zhejiang Provincial Key Laboratory of Plant Evolutionary Ecology and Conservation, Taizhou University, Taizhou 318000, China; 3 School of Forestry, Beijing Forestry University, Beijing 100083, China; 4 The Key Laboratory of Ecological Protection in the Yellow River Basin of National Forestry and Grassland Administration, Beijing Forestry University, Beijing 100083, China

**Keywords:** alligator weed, biomass allocation, DNA methylation, herbivory-induced traits, multiple-generation herbivory effects, root branching order

## Abstract

Interactions between alien plants and local enemies in introduced ranges may determine plant invasion success. However, little is known about whether herbivory-induced responses are transmitted across vegetative generations of plants and whether epigenetic changes are involved during this process. In a greenhouse experiment, we examined the effects of herbivory by the generalist herbivore *Spodoptera litura* on the growth, physiology, biomass allocation and DNA methylation level of the invasive plant *Alternanthera philoxeroides* in the first- (G1), second- (G2) and third-generation (G3). We also tested the effects of root fragments with different branching orders (i.e., the primary- or secondary-root fragments of taproots) of G1 on offspring performance. Our results showed that G1 herbivory promoted the growth of the plants in G2 that sprouted from the secondary-root fragments of G1 but had a neutral or negative effect on the growth of the plants in G2 from the primary-root fragments. The growth of plants in G3 was significantly reduced by G3 herbivory but not affected by G1 herbivory. Plants in G1 exhibited a higher level of DNA methylation when they were damaged by herbivores than when they were not, while neither plants in G2 nor G3 showed herbivory-induced changes in DNA methylation. Overall, the herbivory-induced growth response within one vegetative generation may represent the rapid acclimatization of *A. philoxeroides* to the unpredictable generalist herbivores in the introduced ranges. Herbivory-induced trans-generational effects may be transient for clonal offspring of *A. philoxeroides*, which can be influenced by the branching order of taproots, but be less characterized by DNA methylation.

## 1. Introduction

Biological interactions such as plant-herbivore relationships play a crucial role in the invasion success of at least some alien plants [1,2,3]. The enemy release hypothesis (ERH) postulates that the rapid adaptation and competitive advantage of many alien species in invasive ranges can be attributed to the absence of coevolved native enemies, which greatly contributes to their invasion potential [4]. This hypothesis, despite some support (e.g., [5,6,7]), has also been challenged [1,8,9,10]. For instance, the performance of alien species may still be heavily influenced by local herbivores in the invasive ranges after new plant-herbivore relationships have been established [8,11]. It is, therefore, worthwhile to explore the biological interactions between alien plants and local enemies in invasive ranges, to obtain further insight into the mechanism of plant invasion.

As a common type of local enemy, aboveground insect herbivores often cause direct or indirect harm to plant organs, thereby restricting plant growth and development [12]. For instance, chewing insects such as caterpillars not only severely destroy the physical and physiological structure of plant organs, thereby blocking the mobilization of resources between different organs, but also cause the accelerated senescence of damaged organs through excessive erosion of nutrients [12]. To alleviate herbivory pressure, plants may exhibit a trade-off between tolerance and defense traits. On the one hand, resource allocation often acts as a tolerance trait for damaged plants during their growth and development [13,14,15]. Internal resources (e.g., non-structural carbohydrates) could be mobilized from aboveground damaged plant organs (e.g., leaves and stems) and re-distributed to underground organs [16,17]. Allocation of plant biomass to roots may thus correspondingly increase in damaged plants to prepare for the compensatory growth after damage. On the other hand, as a type of defense trait, the secondary metabolites (e.g., phenolic compounds) may often be selectively released in damaged organs of some species [12,18,19]. The release of chemicals may effectively retard the herbivore feeding rate by either deterring herbivores or reducing the nutritive value of plant tissues [20,21,22]. Due to limitations in resource acquirement, the trade-off between tolerance and defense traits may be crucial to maximizing the fineness of damaged plants under insect herbivory.

Herbivory-induced responses of plants may not only persist within one generation but also transmit across two or more generations. A growing body of two-generation (i.e., parent-offspring pairs) studies has documented that herbivory-induced parental effects could be vital to the fitness of both clonal and non-clonal offspring of plants [16,17,23,24,25,26,27,28,29,30]. In particular, the parental effect is often considered adaptive in sexually or clonally produced offspring since the similarity between parental and offspring environments is predicted to allow offspring to pre-adapt to the predictable environments that parent plants have encountered [31,32,33]. However, there is still uncertainty regarding the stability of herbivory-induced effects over more than two generations of plants, i.e., whether they continue to persist across three generations or even longer [34]. 

DNA methylation is an epigenetic mechanism that drives transgenerational effects in plants [35,36]. DNA methylation can regulate by altering the addition of methyl groups to the nucleotides without changes in the DNA sequences [37]. When environment-induced DNA methylation becomes relatively stable over generations and is less dependent on genetic variation, it can enable offspring to adapt to predictable conditions with some specific strategies [35,38,39,40,41]. Most previous studies have highlighted the role of DNA methylation in the transgenerational effects on shaping defense against and tolerance to herbivory in sexually reproducing plants (e.g., [42,43]), but only a few studies have focused on its role in rapid phenotypic differentiation and local adaptation in some non-model clonal plants such as *Fallopia japonica* [44], and the transgenerational stability and fitness consequences regulated by DNA methylation remain mostly unclear [45,46]. Such knowledge may provide a key insight into the underlying mechanisms of transgenerational effects in clonal plants.

**Figure A1:**
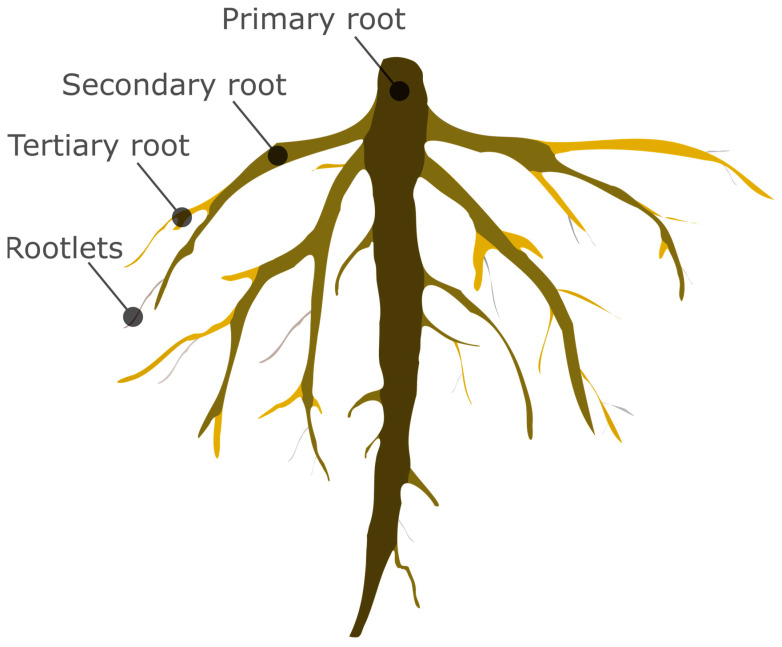
The simplified diagram depicting the branching order of the taproot system of *Alternanthera philoxeroides*. The primary root is the central, first-formed, main root, originating from the clonal fragments; the secondary roots branch off from the primary root.

Roots can be the storage and regeneration organ for many clonal plants [47,48,49]. Root fragments may provide the foundation of clonal offspring when roots can form adventitious buds that will develop into shoots [47,50]. Since roots are a highly branched system organized hierarchically from lower-order distal roots to higher-order basal roots (Figure A1), roots with different orders may differ greatly in structure and function [51,52,53]. One expectation is that the differentiated root vigor, anatomical structure, and chemicals (e.g., non-structural carbohydrates) may remain even after root fragmentation [51] and thereby modify the (grand-)parental effects on clonal offspring originating from root fragments of different orders. However, no study has tested the effects of root fragments with different branching orders on (grand-)parental effects in clonal plants.

We conducted a three-generation experiment on a well-studied, invasive clonal plant, *Alternanthera philoxeroides.* We examined the effects of current, parental, and grand-parental herbivory by the generalist herbivore *Spodoptera litura* on the growth, physiology, and biomass allocation of the first-, second-, and third-generation clonal offspring of *A. philoxeroides*, which were derived from two types of root fragments with different branching orders (i.e., the primary- or secondary-root fragments of the first-generation plants). Additionally, we compared the DNA methylation levels of leaves in different generations between the herbivory treatments. Specially, we tested the following hypotheses: (1) Herbivory effects persist across multiple vegetative generations. In particular, herbivory-induced grand-parental effects will influence the growth, physiology, and biomass allocation of grand offspring. (2) Herbivory-induced transgenerational effects are context-dependent. One expectation is that if grandparent plants experienced herbivory stress, their grand offspring might better respond to herbivory stress due to the inheritance of herbivory-induced traits. Conversely, if grandparent plants have no history of being under herbivory stress, their grand offspring may better respond to the non-herbivory condition without the costs of herbivory-induced traits. (3) Herbivory-induced transgenerational effects differ between clonal offspring sprouted from the root fragments of different orders. (4) Herbivory-induced transgenerational effects in clonal plants are regulated by DNA methylation.

## 2. Results

### 2.1. Performance of Plants in G1

**Figure 1:**
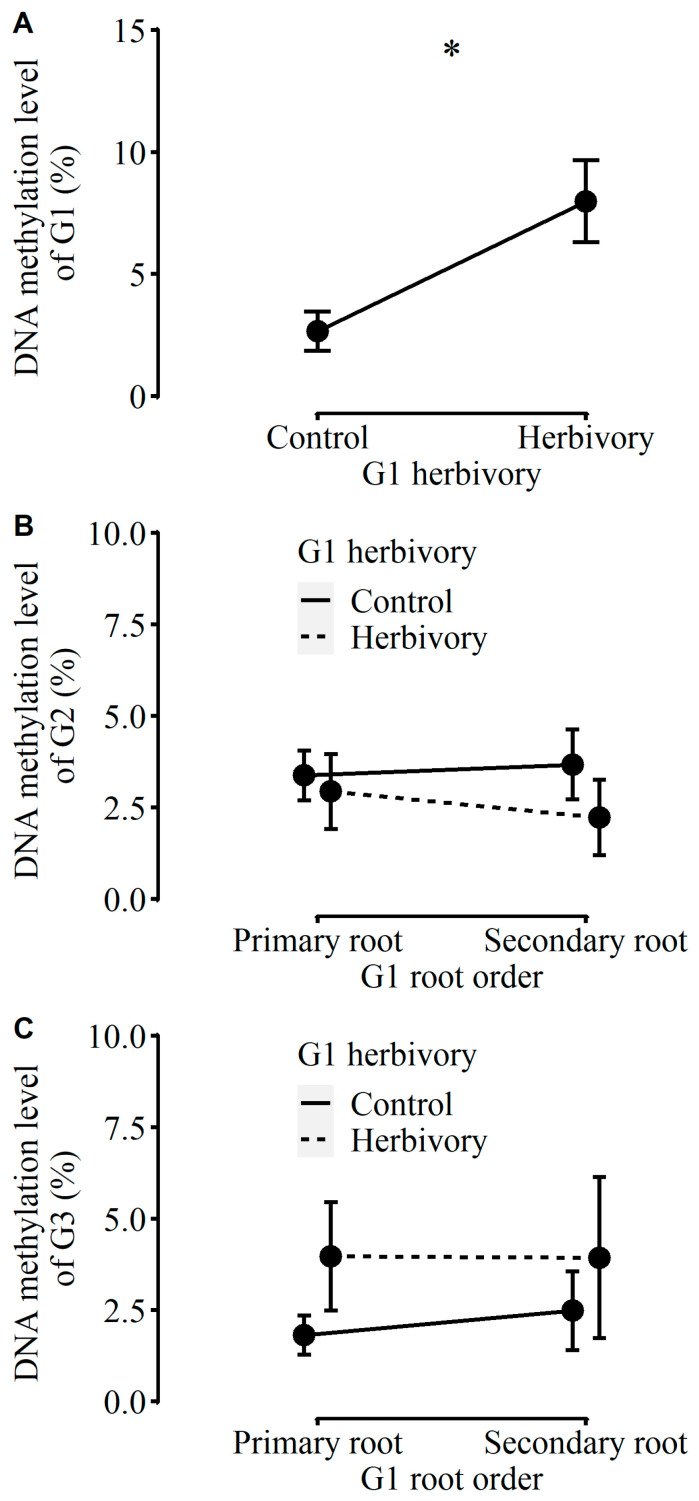
DNA methylation levels of three clonal generations of *Alternanthera philoxeroides*, including (**A**) the DNA methylation level of plants in G1; (**B**) the DNA methylation level of plants in G2; (**C**) the DNA methylation level of plants in G3 without G3 herbivory. In (**B**,**C**), the points and error bars connected by dash lines presented the performance of plants in G2 or G3 taken from G1 with herbivory; the points and error bars connected by solid lines presented the performance of plants in G2 or G3 taken from G1 without herbivory. “*” means a significant difference (*p* < 0.05).

**Figure A2:**
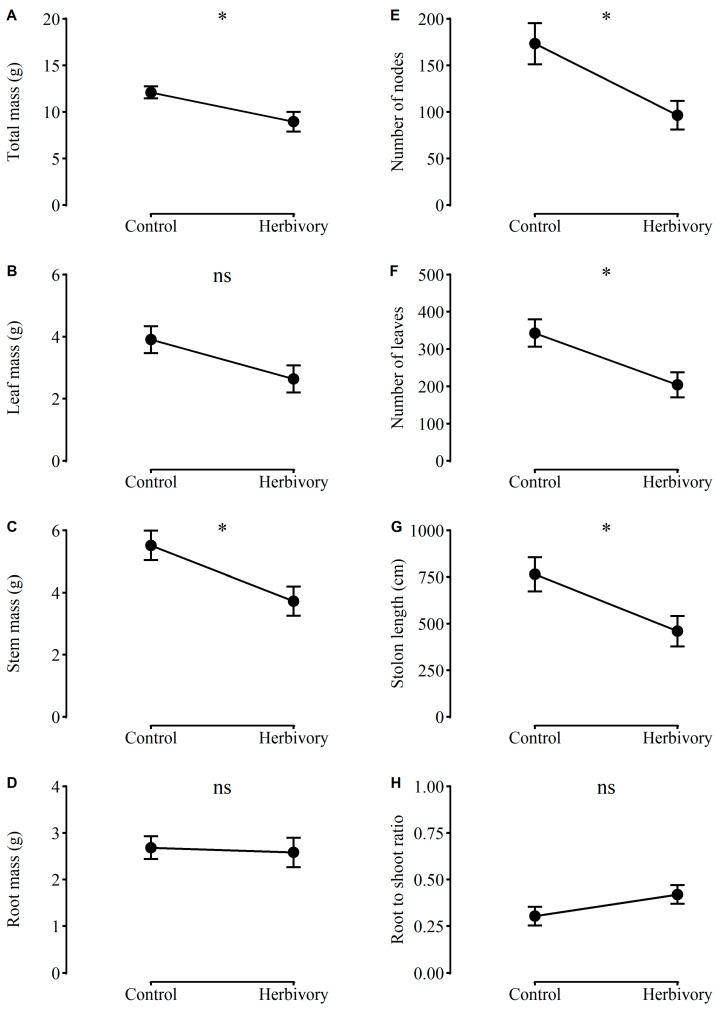
Effects of G1 herbivory on (**A**) total mass, (**B**) leaf mass, (**C**) stem mass, (**D**) root mass, (**E**) number of nodes, (**F**) number of leaves, (**G**) stolon length, and (**H**) root-to-shoot ratio of plants in G1. Degree of freedom (*df*) for herbivory effect is 8. The label “ns” means no significant difference between herbivory treatments; “*” means a significant difference (*p* < 0.05).

**Figure A3:**
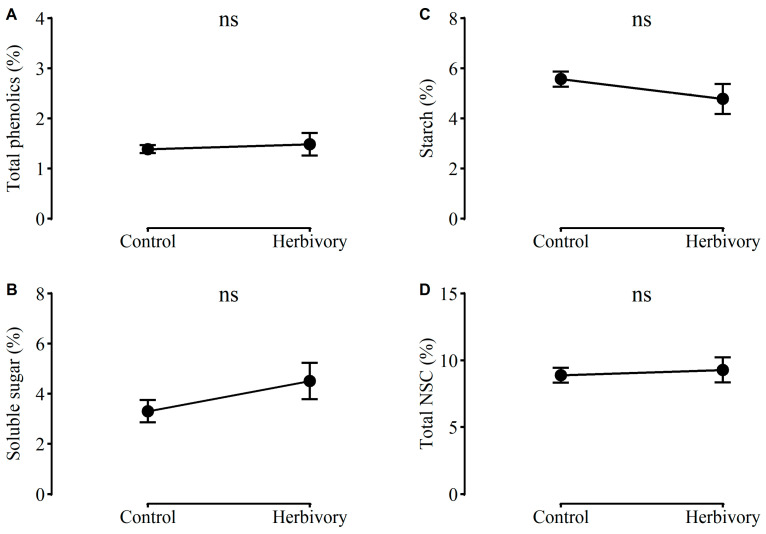
Effects of G1 herbivory on the concentration of (**A**) total phenolic, (**B**) water-soluble sugar, (**C**) starch, and (**D**) total NSC of plants in G1. Degree of freedom (*df*) for herbivory effect is 8. The label “ns” means no significant difference between herbivory treatments.

The total mass, stem mass, number of nodes, number of leaves, and solon length of plants in the first generation (referred to as “G1”) were significantly lower in the herbivory treatment than in the control treatment (Figure A2). Under herbivory, the total mass could be reduced by 26.1% on average. The other growth traits (i.e., leaf mass or root mass) and physiological traits (i.e., the concentration of soluble sugars, starch, total NSC, or total phenolic), as well as biomass allocation (i.e., root-to-shoot ratio), were not affected by herbivory (Figure A2 and Figure A3). By contrast, the DNA methylation level was significantly higher in plants in G1 with herbivory than those without herbivory (2.66% vs. 7.98%; Figure 1A). Furthermore, using the plants in G1 without herbivory and their clonal offspring as the control, we compared them with plants in G1 with herbivory and also found a similar significant difference in DNA methylation levels between each other (2.74% vs. 7.98%; *df* = 25, *p* < 0.001).

### 2.2. Performance of Plants in G2

**Figure 2:**
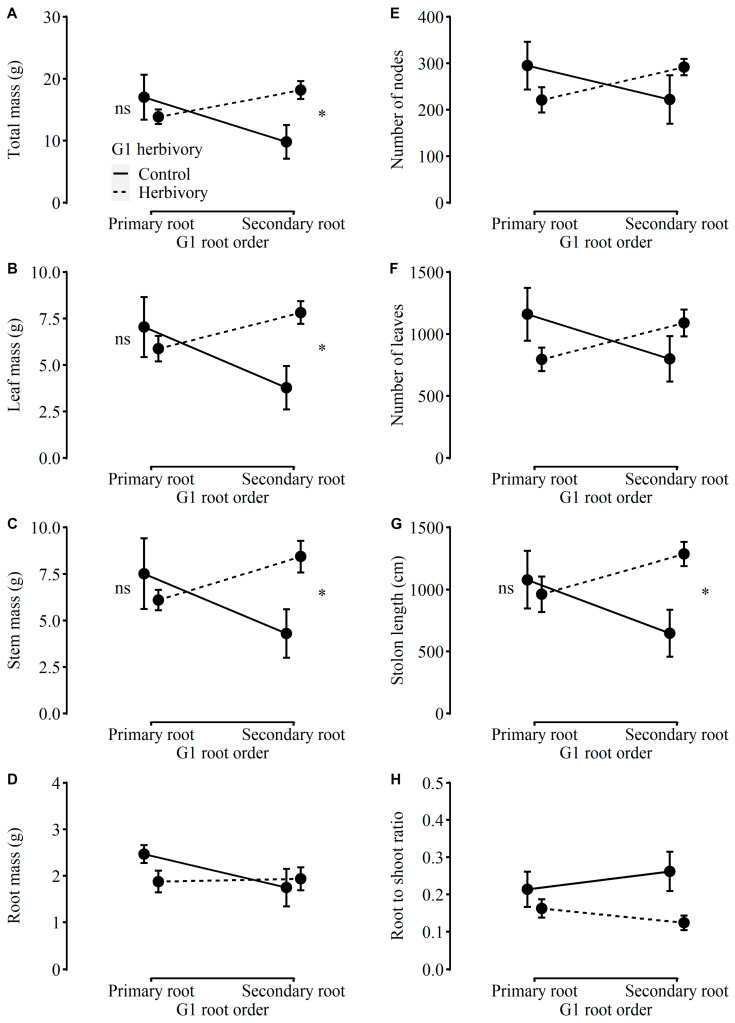
Effects of G1 herbivory and G1 root order on (**A**) total mass, (**B**) leaf mass, (**C**) stem mass, (**D**) root mass, (**E**) number of nodes, (**F**) number of leaves, (**G**) stolon length, and (**H**) root-to-shoot ratio of plants in G2. The label “ns” means no significant difference between G1 herbivory treatments within each root order; “*” means a significant difference (*p* < 0.05).

There were significant interaction effects between the G1 herbivory and G1 root order on the total mass, leaf mass, stem mass, and stolon length of plants in the second generation (referred to as “G2”) (Table 1). The G1 herbivory strongly depended on the root order of G1 (Table 1). The negative effects of G1 herbivory on the total mass, leaf mass, stem mass, and stolon length were weak and not significant in plants in G2 taken from primary-root fragments of G1, while the positive effects of G1 herbivory were significantly detected in plants in G2 taken from secondary-root fragments of G1 (Figure 2A–C,G). Similarly, the MANOVA results indicated that G1 herbivory significantly negatively influenced growth traits, although the effects of G1 root order and their interactions were not significant (Table A1).

**Figure 3:**
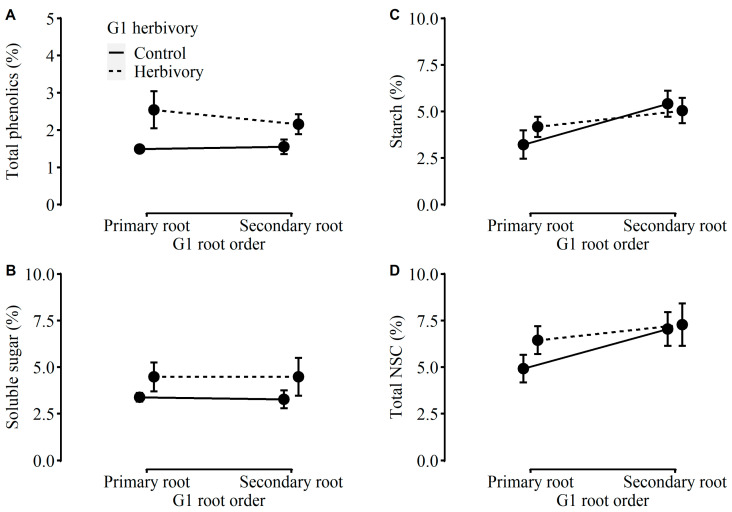
Effects of G1 herbivory and G1 root order on the concentration of (**A**) total phenolic, (**B**) water-soluble sugar, (**C**) starch, and (**D**) total NSC of plants in G2.

The root-to-shoot ratio and the concentration of total phenolic were independently affected by G1 herbivory (Table 1). Compared to plants in G2 produced by G1 without herbivory, plants in G2 produced by G1 with herbivory had a lower root-to-shoot ratio and a higher concentration of total phenolic (Figure 2H and Figure 3A). Otherwise, the other growth traits (i.e., root mass, number of nodes, or number of leaves), physiological traits (i.e., the concentrations of water-soluble sugars, starch, or total NSC), or DNA methylation levels were influenced by neither G1 herbivory nor G1 root order (Table 1, Figure 1B, Figure 2 and Figure 3). Indeed, the MANOVA results indicated that there were no significant main or interaction effects on physiological traits (Table A1).

### 2.3. Performance of Plants in G3

**Figure 4:**
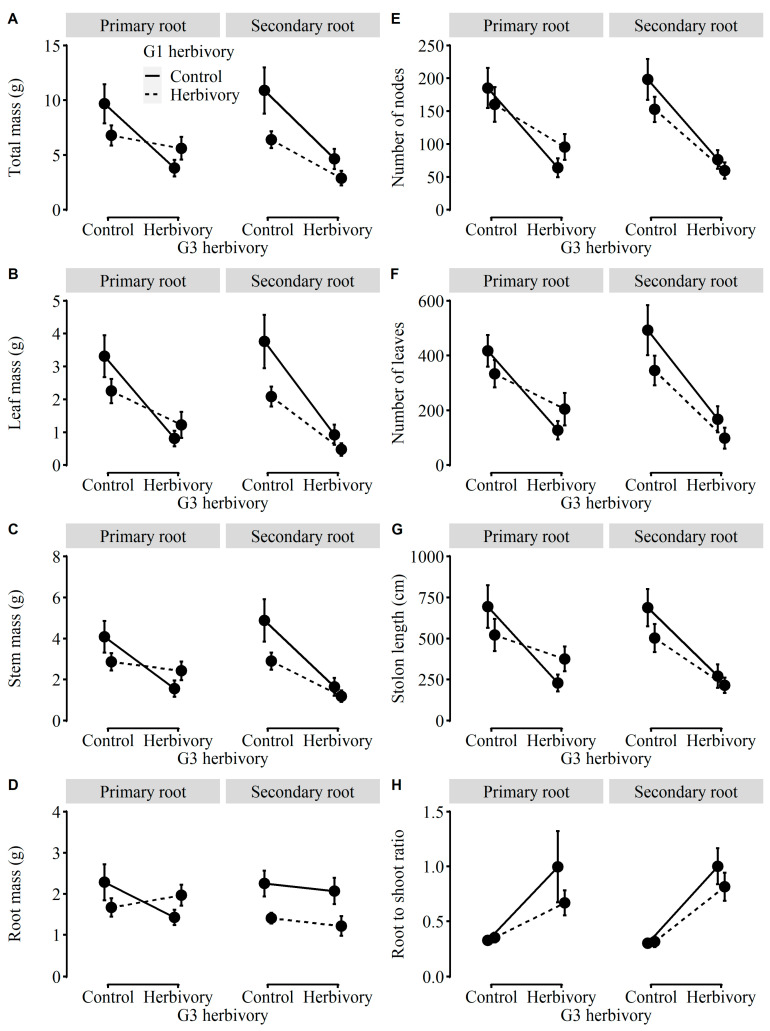
Effects of G1 herbivory, G1 root order, and G3 herbivory on (**A**) total mass, (**B**) leaf mass, (**C**) stem mass, (**D**) root mass, (**E**) number of nodes, (**F**) number of leaves, (**G**) stolon length, and (**H**) root-to-shoot ratio of plants in G3.

**Figure 5:**
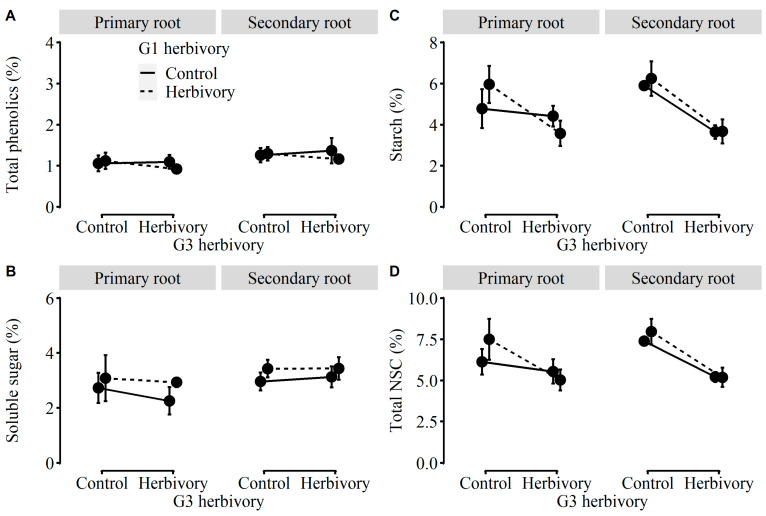
Effects of G1 herbivory, G1 root order, and G3 herbivory on the concentrations of (**A**) total phenolic, (**B**) water-soluble sugar, (**C**) starch, and (**D**) total NSC of plants in G3.

Being consistent with the effects of herbivory on plants in G1, most of the growth traits (i.e., total mass, leaf mass, stem mass, number of nodes, number of leaves, and stolon length), physiological traits (i.e., the contractions of starch and total NSC), and biomass allocation (i.e., root-to-shoot ratio) of plants in the third generation (referred as to “G3”) were strongly influenced by G3 herbivory (Table 2). The total mass, leaf mass, stem mass, number of nodes, number of leaves, stolon length, and the concentrations of starch and total NSC were significantly lower in plants in G3 with G3 herbivory than without G3 herbivory (Figure 4 and Figure 5). Under herbivory, the total mass could be reduced by 49.8% on average. On the contrary, the root-to-shoot ratio was significantly higher in plants in G3 with G3 herbivory than without G3 herbivory (Figure 4H). Likewise, the MANOVA results showed that only G3 herbivory significantly inhibited the growth and physiological traits of plants in G3 (Table A2).

Only the total mass, leaf mass, and root mass of plants in G3 tended to be or were significantly influenced by G1 herbivory (Table 2). The total mass, leaf mass, and root mass were relatively higher in plants in G3 derived from G1 without herbivory than those from G1 with herbivory (Figure 4A,B,D). There were no significant main effects caused by the root order of G1 (Table 2). However, the negative effects of G1 herbivory on root mass were significant in plants in G3 derived from the secondary-root fragments of G1 (*p* = 0.003) but weakened in those from the primary-root fragments of G1 (*p* = 0.900; Table 2, Figure 4D). 

Apart from root mass, there were no other two-way or three-way interaction effects on growth and physiological traits or biomass allocation (Table 2). Moreover, there were no significant main and interaction effects on the concentrations of total phenolic and soluble sugars of plants in G3 (Table 2, Figure 5) and no effects on the DNA methylation levels of plants in G3 without current herbivory (Figure 1C, Table 2).

## 3. Discussion

### 3.1. Direct Herbivory Effects in G1 and G3

For growth traits, irrespective of plants in G1 and G3, the aboveground herbivory caused by larvae of *Spodoptera litura* could directly postpone and retard the development of the leaf organ, thereby leading to a dramatic reduction in the aboveground growth and overall fitness of the damaged plants of *A. philoxeroides.* In contrast, the aboveground herbivory had no direct impact on the root growth of the damaged plants, so a relatively higher root-to-shoot ratio was found in damaged plants. Several previous studies have also shown that damaged plants might allocate more biomass to underground organs when aboveground organs were completely or partially removed to actively compensate for the loss of aboveground organs and preserve storage resources for regrowth under favorable conditions [16,54,55]. However, a higher root-to-shoot ratio might also be a byproduct of aboveground removal rather than a differential response. The latter may be further determined by comparing root growth rates after imposing damage between control and herbivory treatments.

For physiological traits, the aboveground herbivory had little impact on the concentrations of primary and secondary metabolites of leaves in the damaged plants, except for the decreased concentration of leaf starch and non-structural carbohydrates in plants in G3. The results suggested that *A. philoxeroides* did not appear to directly defend against the attack of aboveground herbivores such as *S. litura* with the release of leaf chemicals but instead tolerant to the aboveground herbivory via the mobilization and maintenance of internal resources in roots [16,17]. The latter strategy might better facilitate the rapid regrowth and vegetative reproduction of plants after herbivore damage to compensate for herbivory loss [56,57,58]. Meanwhile, such tolerance traits might greatly contribute to the potential invasion of *A. philoxeroides* in the invasive ranges, especially when they are confronted with unpredictable generalist herbivores in the new habitats [59,60,61].

### 3.2. Transgenerational Effects in G2 and G3

For plants in G2, the root order of G1 (i.e., primary and secondary roots) was included in the analyses. Interestingly, the G1’s history of being damaged by *S. litura* could positively influence the aboveground performance of plants in G2 originating from secondary-root fragments but had neutral impacts on the performance of plants in G2 originating from primary-root fragments. In detail, compared to the clonal plants in G2 taken from the secondary-root fragments of G1 without herbivory, the clonal plants in G2 taken from the secondary-root fragments of G1 with herbivory, had better aboveground growth and stolon expansion ability (Table 1). On the other hand, regardless of the root order, plants in G1 that experienced herbivory both allowed plants in G2 to have the capacity to accumulate more phenolic in leaves and maintain a high biomass allocation to roots. These results supported the first hypothesis, suggesting that herbivory-induced parental effects via vegetative reproduction could be transmitted between two vegetative generations, which contributed to the establishment of herbivory-induced traits in clonal offspring. Such herbivory-induced transgenerational effects were also observed in terrestrial clonal plants such as *Leymus chinensis* [62] and *Solanum carolinense* [28]. For instance, a recent study found that clonal offspring of *L. chinensis* from grazed sites displayed transgenerational trait plasticity in terms of herbivory-avoidance traits (e.g., reduced height but greater tiller density) to maintain high aboveground production [62].

Also, as predicted by the third hypothesis, such G1 effects on aboveground performance could depend on the root order of plants in G1, from which plants in G2 are produced. However, the performance difference between clonal offspring from roots of different orders did not appear to be associated with their initial size because the primary and secondary root fragments used in the G2 experiment were 7.7 mg and 13.0 mg fresh mass (personal observation), respectively [17]. Alternatively, we speculated that when plants of *A. philoxeroides* are given damage, secondary root fragments may be able to germinate and grow more vigorously than primary roots, resulting in higher growth rates of clonal offspring [51,53]. Future studies could reveal the mechanism underlying their differential growth by comparing the quality between roots of different orders. Nevertheless, this may be the first report demonstrating the influence of root orders on parental effects in clonal plants.

For plants in G3, the G1 herbivory effects on plant characteristics related to growth, physiology, and biomass allocation were mostly neutral, and they did not interact with the current herbivory environment. Only the root growth of plants in G3, derived from the secondary fragments of plants in G1, was negatively affected due to G1’s history of being damaged by *S. litura*, suggesting that maladaptive transgenerational effects existed, but they were limited in some specific organs of clonal offspring with the similar experience of being damaged. The overall results were inconsistent with the second hypothesis and indicated that the persistence of herbivory-induced responses across vegetative generations was not stable for *A. philoxeroides*. One plausible reason is that herbivory-induced traits tend to function as a local acclimation of *A. philoxeroides* to local generalist enemies in the introduced ranges, resulting in local phenotypic plasticity as opposed to transgenerational inheritance [63,64]. A recent study on *Carpobrotus edulis* also documented that phenotypic plasticity may contribute to the successful and rapid adaptation of this species to new habitats, but these phenotypic changes seem to be independent of epigenetic ones [65]. The other plausible reason is that the historical, biological interactions between invasive plants and their native coevolved enemies may be the foundation for the adaptive herbivory-induced responses that pass across generations [13,66]. If so, some additional studies will have to be undertaken to determine whether transgenerational effects differ in clonal plants when they encounter generalist and specialist herbivores.

### 3.3. Role of DNA Methylation

DNA methylation can be modulated by environmental stress and plays an important role in the phenotypic plasticity of environmental changes [35,38,39,40,41,44]. In our work, for plants in G1, the aboveground herbivory triggered a dramatic increase (about three times higher) in the DNA methylation levels of the damaged leaves (Figure 1A). This may imply that the herbivory-induced growth responses within one generation could be closely associated with some chromatin modifications (i.e., DNA methylation). The findings are also consistent with previous studies that have examined the role of epigenetic changes in *A. philoxeroides* under other environmental factors, such as flooding stress, suggesting that epigenetic variation may be a consequence of environmental induction and spontaneous epimutation [67,68]. In a recent study of the association between insect herbivory and DNA methylation, it was also documented that the defense traits (including physical and chemical traits) of the perennial herbaceous species *Raphanus sativus* induced by aboveground herbivory of caterpillars could correspond to the increase in the methylation probabilities in DNA sequences of the damaged plants [30]. Inconsistent with the fourth hypothesis, as with no obvious herbivory-induced transgenerational effects in G2 and G3 (with the exception of plants in G2 and G3 sprouted from the secondary roots of G1), herbivory-induced methylation of DNA may easily drop to an average level (between 2.2% and 4.0%) when vegetative generations are continuously established. These results further underscored that herbivory-induced transgenerational plasticity might not be a promising strategy for *A*. *philoxeroides* to adapt to local enemies in the introduced ranges.

## 4. Materials and Methods

### 4.1. Study Species

*Alternanthera philoxeroides* (Mart.) Griseb., native to South America, is a creeping perennial herb of the Amaranthaceae family [69]. The species was intentionally introduced into China as livestock feed in the 1930s and is now considered one of the most noxious invasive weeds [70]. The genetic diversity of *A. philoxeroides* is extremely low in China, and most individuals are recruited by clonal growth [71,72]. Fragmentation of stolons and roots became the primary means for *A. philoxeroides* to produce vegetative offspring and to self-maintain their field populations [17,57]. *Spodoptera litura* Fabricius belongs to the Noctuidae family, which is distributed all over the world. The species is an omnivorous agricultural pest and is also reported as a generalist herbivore of *A. philoxeroides* in the field [61,73].

In May 2011, plants of *A. philoxeroides* were collected from a riparian agricultural area (28.87° N, 121.01° E) in Taizhou, Zhejiang Province, China. They were vegetatively propagated in the greenhouse at Forest Science Co., Ltd. (Guangzhou, China), at Beijing Forestry University. Larvae of *S. litura* were obtained from Henan Jiyuan Baiyun Industrial Co., Ltd. (Jiyuan, China) (http://www.keyunnpv.cn/ (accessed on 18–22 August 2018). The mean air temperature in the greenhouse during the experiment series was 21.28 ± 0.36 °C (mean ± SE), as measured with a HOBO Pendant Temperature/Light Data Logger (UA-002-64; Onset Computer, Bourne, MA, USA).

### 4.2. Experimental Design

**Figure 6:**
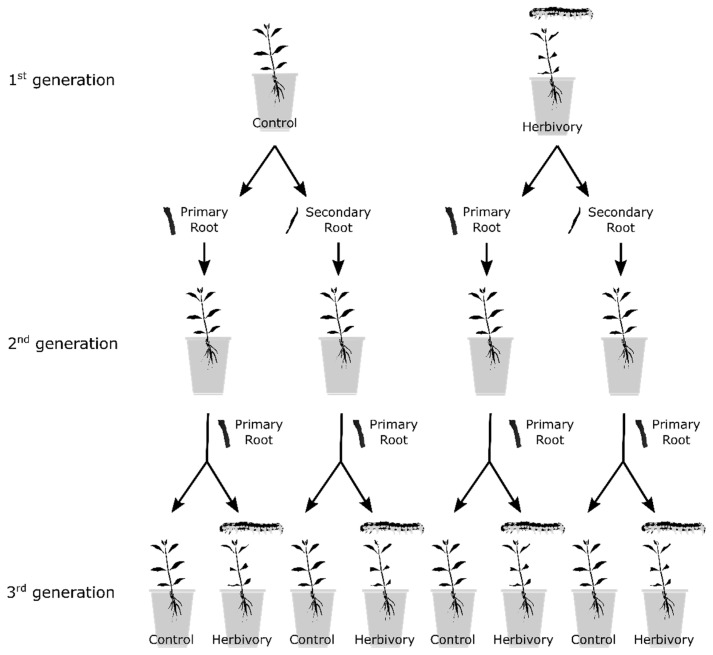
The diagram of the experimental design. Three clonal generations of *Alternanthera philoxeroides* were used in the experiment. In the first generation (G1), plants were grown without or with herbivory (control vs. herbivory by *Spodoptera litura*). In the second generation (G2), plants were subjected to two-way factorial treatments, including G1 herbivory (i.e., control vs. herbivory in G1) and G1 root order (i.e., primary or secondary roots of G1). In the third generation (G3), plants were subjected to three-way factorial treatments, including G1 herbivory, G1 root order, and G3 herbivory (i.e., control vs. herbivory in G3).

Three clonal generations of *A. philoxeroides* were used in the experiment series (Figure 6). In order to simplify the terminology, the first generation of plants was referred to as “G1”, the second generation as “G2”, and the third generation as “G3”. On 13 May 2018, 840 stem fragments, each with one stem node, two internodes 6 cm long, and two opposite leaves, were cut off from the stock plants of *A. philoxeroides*. They were grown in 50 × 50 × 8 cm containers (long × wide × high) filled with a mixture of commercial nutrient solution (Scotts Miracle-Gro All Purpose; Scotts Miracle-Gro Company, Wuhan, China) and tap water at a ratio of 1:16 for one month. During the 40-day cultivation, a shoot started to come out from one axillary bud of the stem node, and then roots started to develop (hereafter, the rooted stem node with a shoot is called a plant). These plants were used for the G1–G3 experiments. The G1 experiment was conducted to establish the herbivory condition in the first generation; the G2 experiment was conducted to test hypotheses 1–4 in the second generation; the G3 experiment was conducted to test hypotheses 1–4 in the third generation.

**G1 experiment:** On 21 June 2018, a subset of 48 plants with uniform sizes were transplanted into plastic pots (14 cm in diameter and 12 cm deep) and cultivated for two months. The pots were filled with a mixture of clay soil and peat (Pindstrup Seedling; Pindstrup Mosebrug A/S, Ryomgaard, Denmark) at a volume ratio of 1:1 (total P in average: 0.47 mg/g; total N: 1.62 mg/g). On 23 August 2018, these plants in G1 were randomly assigned to one of two herbivory treatments, i.e., without herbivory (control) or with herbivory by *S. litura*. Each treatment was replicated 24 times. In the herbivory treatment, eight fourth-instar larvae of *S. litura* were released on the leaf surface of each plant for two weeks and then removed. After two weeks of recovery, another eight fourth-instar larvae of *S. litura* were released on the leaf surface of each plant for another two weeks and then removed. Two weeks after completely stopping grazing, on 4 October 2018, five replicates of the plants were randomly selected and then harvested to measure biomass and offspring ramet production. For the remaining 19 replicates of the plants, primary and secondary root fragments with a 6 cm length were harvested and used for the second-generation experiment. The initial fresh masses of the primary and secondary root fragments produced by plants in G1 without herbivory were 1.37 ± 0.23 mg and 0.86 ± 0.15 mg (mean ± SE), respectively; the initial fresh masses of the primary and secondary root fragments produced by plants in G1 with herbivory were 1.24 ± 0.06 mg and 0.68 ± 0.07 mg, respectively.

**G2 experiment:** Plants in G2 developed from the primary root, and the secondary root fragments produced by plants in G1 subjected to herbivory by *S. litura* or not (Figure A1) were grown isolated from herbivory. Thus, the G2 experiment consisted of two factors, i.e., G1 root order (primary vs. secondary root fragments produced by plants in G1) and G1 herbivory (plants in G1 with or without herbivory).

These primary and secondary root fragments were planted in pots of the same size and filled with the same soil mixture as described in the first-generation experiment. The G2 experiment was initiated shortly after the G1 experiment was harvested and lasted for about eight months. On 22–23 June 2019, five replicates of plants in G2 sprouted from the root fragments of each of the four types were harvested. For the remaining 14 replicates of plants in G2, primary root fragments with a 6 cm length were harvested and used for the G3 experiment.

**G3 experiment**: Plants in G3 developed from the root fragments produced by plants in G2, and each of the four types was again subjected to herbivory by *S. litura* or not (Figure A1). Thus, the G3 experiment consisted of three factors, i.e., G1 root order, G1 herbivory, and G3 herbivory (plants in G3 with or without herbivory).

Again, the root fragments of plants in G2 were planted in pots of the same size and filled with the same soil mixture as described in the G1 experiment. The G3 experiment was also initiated shortly after the G2 experiment was harvested. The root fragments were allowed to germinate and produce shoots of plants in G3 without herbivory in the first two months. On 26 August 2019, plants in G3 were randomly assigned to one of two herbivory treatments, i.e., without herbivory (control) or with herbivory by *S. litura*. Herbivory treatment followed the same procedure as in the G1 experiment. Seven replicates of plants in G3 per each of the third-generation treatments were harvested.

### 4.3. Measurements

For three clonal generations of *A. philoxeroides,* the number of stem nodes, the number of leaves, and the stolon length were measured after each generation harvest. Four of the youngest fresh leaves for plants in each generation were stored in plastic bags filled with silica gel for the measurement of DNA methylation levels. Unfortunately, the youngest leaves of plants in G3 with G3 herbivory were not collected for the further measurement of DNA methylation levels due to the severe destruction of the youngest leaves caused by the herbivory of *S*. *litura*. The remaining leaves, stems, and roots of each plant were separated, dried in an oven at 70 °C for 48 h, and weighed.

Leaves of *A. philoxeroides* dried in the oven were finely ground using a Retsch MM400 Mixer Mill at a frequency of 4000 Hz for 10 min (Retsch GmbH, Haan, Germany) and used for the measurement of physiological traits. The concentrations of non-structural carbohydrate compounds (hereafter named “NSC”) in leaves (including water-soluble sugars and starch) were measured using the perchloric acid/anthrone method, and the concentration of total phenolic in leaves was measured with the Folin-Ciocalteu method (see details in [16,17,25]). Physiological measurements were replicated five times for plants in G1 and G3 and four times for plants in G2.

For the measurement of DNA methylation levels, the total genome DNA from each silica gel-dried leaf sample was first isolated following the manual of the TIANGAN Plant Genomic DNA kit (DP350; Tiangen Biotech, Beijing, China). The global DNA methylation levels in leaves were then quantified with the MethylFlash™ Global DNA Methylation (5-mC) ELISA Easy Kit (Colorimetric) (AP1030; Epigentek Group Inc., Farmingdale, NY, USA). The MethylFlash kit uses an enzyme-linked immunosorbent assay (ELISA) to estimate the global DNA methylation (%5-mc) by measuring an amount of 5-methylcytosine (5-mC) from the DNA extracts. This method of global DNA methylation quantification has been increasingly used in plant species [74,75,76]. DNA methylation measurements were replicated five times for plants in G1 and G3 without herbivory and four times for plants in G1 with herbivory, and for plants in G2.

### 4.4. Data Analysis

For plants in G1, independent t-tests were employed to test the effects of herbivory on growth traits (i.e., total mass, leaf mass, stem mass, root mass, number of stem nodes, and number of leaves), biomass allocation (root-to-shoot ratio), physiological traits (the concentrations of water-soluble sugar, starch, total non-structural carbohydrates [i.e., the summed concentration of water-soluble sugar and starch], and total phenolic), and the DNA methylation level. For plants in G2, two-way ANOVAs were used to test the effects of the parental herbivory, parental root order, and their interaction on plant growth, biomass allocation, physiological traits, and DNA methylation level. For plants in G3, three-way ANOVAs were used to test the effects of the G1 herbivory, G1 root order, and G3 herbivory and their interactions on plant growth, biomass allocation, and physiological traits. For lack of the youngest leaves of plants in G3 with G3 herbivory, we only used the two-way ANOVA to examine the effects of the G1 herbivory, G1 root order, and their interaction on DNA methylation level of plants in G3 without herbivory. Linear contrasts were followed when significant interactions were detected. For plants in G2 and G3, MANOVAs were employed to test the main and interaction effects on growth traits (excluding total mass) and physiological traits. If the data did not meet the assumptions of normality and homogeneity of variance, they were square-root transformed. All analyses were conducted using R v 4.0.5 [77].

## 5. Conclusions

The damage of generalist herbivores such as *S. litura* significantly inhibited the performance of *A. philoxeroides* in the invasive ranges. To reduce herbivory loss, tolerance traits (e.g., biomass allocation to roots) rather than defense traits (e.g., the release of phenolic) became the primary strategy of the introduced *A. philoxeroides*. These herbivory-induced responses may further benefit the development of clonally produced offspring, but these effects are closely related to the root order of parent plants. It is also worth noting that the herbivory-induced responses may not always be stable across vegetative generations, and most of them could terminate after two generations of our study species. This decline of transgenerational effects also corresponds to the stabilized DNA methylation levels in the second and third generations. This also suggests that within-generation phenotypic plasticity, rather than transgenerational inheritance, may provide a more efficient means for *A. philoxeroides* to acclimate to the unpredictable herbivory environments by generalist enemies in the invasive ranges. Besides, since the developmental timing of previous generations might potentially influence the formation of transgenerational signals in subsequent generations, this potential impact could be considered in future experiments.

## Figures and Tables

**Table 1:** Results of ANOVAs for plants in G2. Two-way ANOVAs were employed to test effects of G1 herbivory and G1 root order on growth traits, biomass allocation, physiological traits, and DNA methylation levels of plants in G2.

Variables	G1 Herbivory (G1H)		G1 Root Order (GR)		G1H × GR	
	*F*	*p*	*F*	*p*	*F*	*p*
**Growth**						
Total mass	1.13	0.303	0.35	0.565	**5.53**	**0.032**
Leaf mass	1.74	0.206	0.37	0.553	**5.65**	**0.030**
Stem mass	1.17	0.295	0.13	0.727	**4.86**	**0.043**
Root mass	0.50	0.488	1.37	0.259	1.91	0.185
Number of nodes	<0.01	0.963	<0.01	0.978	3.23	0.091
Number of leaves	0.05	0.818	0.04	0.837	4.31	0.054
Solon length	2.28	0.151	0.10	0.761	**4.77**	**0.044**
**Biomass allocation**						
Root-to-shoot ratio	**5.99**	**0.026**	0.01	0.905	1.25	0.280
**Physiology**						
Total phenolic	**7.50**	**0.018**	0.29	0.600	0.54	0.477
Water-soluble sugar	2.75	0.123	0.01	0.939	0.01	0.936
Starch	0.19	0.674	5.13	0.043	0.95	0.349
Total NSC	0.95	0.348	2.68	0.127	0.52	0.486
DNA methylation	1.02	0.332	0.05	0.826	0.30	0.597

Degrees of freedom (*df*) for effects are 1, 16 for growth traits and biomass allocation and 1, 12 for physiological traits and DNA methylation, respectively. Values for which *p* < 0.05 are in bold.

**Table 2:** Results of ANOVAs for plants in G3. Two-way or three-way ANOVAs were employed to test effects of G1 herbivory, G1 root order, or G3 herbivory on growth traits, biomass allocation, physiological traits, and DNA methylation levels of plants in G3.

Variable	G1 Herbivory (G1H)		G1 Root Order (GR)			G3 Herbivory (G3H)		G1H × GR		G1H × G3H		GR × G3H		G1H × GR × G3H	
	*F*	*p*	*F*	*p*		*F*	*p*	*F*	*p*	*F*	*p*	*F*	*p*	*F*	*p*
Growth															
Total mass	3.18	0.081	0.26	0.614		**25.94**	**<0.001**	2.96	0.092	2.78	0.102	0.79	0.380	0.93	0.339
Leaf mass	3.12	0.084	0.51	0.479		**49.52**	**<0.001**	1.85	0.180	2.08	0.156	0.95	0.334	0.46	0.502
Stem mass	1.52	0.223	0.19	0.665		**26.46**	**<0.001**	1.99	0.165	3.50	0.067	1.53	0.222	0.55	0.462
Root mass	**5.10**	**0.029**	0.26	0.610		1.40	0.243	**4.30**	**0.044**	2.14	0.150	0.05	0.816	2.20	0.144
Number of nodes	0.80	0.376	0.08	0.776		**41.13**	**<0.001**	1.22	0.275	1.85	0.180	0.22	0.645	0.20	0.655
Number of leaves	1.96	0.168	0.02	0.896		**38.56**	**<0.001**	1.72	0.195	2.24	0.141	0.93	0.340	0.26	0.610
Stolon length	1.16	0.288	0.33	0.567		**27.90**	**<0.001**	0.75	0.392	3.27	0.077	0.14	0.713	0.59	0.447
Biomass allocation															
Root-to-shoot ratio	1.37	0.248	0.05	0.833		**28.50**	**<0.001**	0.10	0.756	1.85	0.180	0.28	0.602	0.15	0.705
Physiology															
Total phenolic	0.27	0.606	2.78	0.105		0.11	0.737	0.01	0.911	0.79	0.381	0.07	0.791	<0.01	0.996
water-soluble sugar	1.79	0.191	2.13	0.154		0.12	0.736	0.04	0.850	0.01	0.908	0.35	0.556	0.13	0.722
Starch	0.15	0.706	0.15	0.698		**16.29**	**<0.001**	<0.01	0.985	1.55	0.222	1.23	0.276	0.82	0.371
Total NSC	0.47	0.498	0.57	0.457		**15.26**	**<0.001**	0.02	0.884	1.48	0.233	0.85	0.363	0.40	0.533
DNA methylation	1.54	0.233	0.05	0.831		/	/	0.06	0.813	/	/	/	/	/	/

Degrees of freedom (*df*) for effects are 1, 48 for growth traits and biomass allocation, 1, 32 for physiological traits, and 1, 16 for DNA methylation, respectively. Total mass, leaf mass, and stem mass were square-root transformed. Values for which *p* < 0.05 are in bold.

## Data Availability

Raw data and R codes for the main analyses are deposited in a publicly accessible GitHub repository (https://github.com/bichengdong/Transgenerational_herbivory_effects).

## Appendix A

**Table A1:** Results of MANOVAs for plants in G2. Two-way MANOVAs were employed to test effects of G1 herbivory and G1 root order on growth traits and physiological traits of plants in G2.

	Growth Traits		Physiological Traits	
	*F*	*p*	*F*	*p*
G1 herbivory (G1H)	**5.91**	**0.006**	1.41	0.305
G1 root order (GR)	1.80	0.188	2.91	0.084
G1H × GR	1.57	0.244	0.82	0.546

Degrees of freedom (*df*) effects are 1, 11 for growth traits and 1, 9 for physiological traits, respectively. Total mass was excluded from the growth traits in MANOVA. Values for which *p* < 0.05 are in bold.

**Table A2:** Results of MANOVAs for plants in G3. Three-way MANOVAs were employed to test effects of G1 herbivory, G1 root order, and G3 herbivory on growth traits and physiological traits of plants in G3.

	Growth Traits		Physiological Traits	
	*F*	*p*	*F*	*p*
G1 herbivory (G1H)	1.13	0.364	0.67	0.616
G1 root order (GR)	0.99	0.446	0.99	0.431
G3 herbivory (G3H)	**17.77**	**<0.001**	**4.44**	**0.006**
G1H × GR	0.90	0.507	0.33	0.855
G1H × G3H	0.85	0.536	0.93	0.463
GR × G3H	1.69	0.148	0.73	0.582
G1H × GR × G3H	0.61	0.723	0.43	0.783

Degrees of freedom (*df*) for effects are 1, 43 for growth traits and 1, 29 for physiological traits, respectively. Total mass was excluded from the growth traits in MANOVA. Leaf mass and stem mass were square-root transformed. Values for which *p* < 0.05 are in bold.
